# Cancer risk in patients with four non-specific symptoms: a decade of research and implications for practice

**DOI:** 10.3399/BJGP.2026.0088

**Published:** 2026-10-01

**Authors:** Charlotte Williamson, Anjali Behal, Becky White, Meena Rafiq, Samantha Harrison, Brian D Nicholson

**Affiliations:** 1 Cancer Research UK, London, UK; 2 Epidemiology of Cancer Healthcare and Outcomes Group, Department of Behavioural Science, Institute of Epidemiology and Health Care, University College London, London, UK; 3 Department of General Practice and Primary Care, University of Melbourne, Melbourne, Australia; 4 Nuffield Department of Primary Care Health Sciences, University of Oxford, Oxford, UK

In England, the most common route to cancer diagnosis is via general practice,^1^ with similar patterns seen across the UK and similar international jurisdictions.^2^ Timely recognition of suspected cancers in primary care, appropriate testing, and referral along managed urgent cancer pathways can improve patient outcomes through earlier diagnosis.^3^ Often, site-specific alarm symptoms, for example, haemoptysis for lung cancer, have a clear referral pathway. However, over half of patients diagnosed with cancer present with non-specific symptoms (NSS).^4,5^ These include unexplained weight loss, fatigue, abdominal symptoms, nausea, vomiting, appetite loss, and non-specific pain. Determining onward management of this patient group poses a challenge for primary care.

This analysis aims to outline the challenges of NSS management, critically appraise research from the past decade related to four common NSS (unexplained weight loss, abdominal pain, bloating, and fatigue), and consider the opportunities for the future management of NSS.

## The challenge

NSS are usually associated with a lower risk for individual cancer sites than site-specific alarm symptoms. Patients diagnosed with cancer who present with NSS experience prolonged intervals from presentation to diagnosis, later-stage diagnosis, and diagnosis via emergency presentation.^6^

National cancer referral guidelines, such as the National Institute for Health and Care Excellence (NICE) NG12 used in England and Wales, employ cancer risk thresholds based on signs and symptoms. NICE NG12 generally employs a 3% threshold, which means that patients with a cancer risk above this threshold should be considered for urgent specialist investigation or referral. Non-specific symptoms often will not reach this threshold for referral.^7^

The NICE NG12 guideline was comprehensively updated in 2015, at which time there was little research investigating cancer risk in people presenting with NSS, and no managed care pathways for this cohort in the UK. There has been much research and service development over the past decade to address this challenge.

NSS pathways have been implemented across the UK to provide a route for GPs to refer patients exhibiting NSS onto a managed pathway. England’s national NSS pathway launched in 2019 and completed rollout in 2024. Similar pathways exist across Wales (rapid diagnostic clinics), Scotland (rapid cancer diagnostic services), and Northern Ireland (rapid diagnostic centres). Pathways in the UK were based on a Danish model that demonstrated timelier diagnosis of cancer and other serious conditions.^4,8,9^

Across the UK there are significant challenges to optimal implementation of these pathways, including variation in how pathways are designed, organised, and funded, with varied levels of engagement from primary and secondary care.^10^

## Non-specific symptoms pathway evaluation

Metrics such as cancer conversion rate, time to diagnosis, cancer site and stage, the proportion of non-cancer diagnoses, and qualitative insights from patients and staff have been used to evaluate the pathways in some areas of the UK. Key metrics such as number of GP consultations prior to referral and emergency diagnoses of cancer avoided have not been explored, nor the impact of the pathways compared with a control group. Despite these limitations, evaluations across the UK highlight the benefits of the pathway for patients, through comparable cancer conversion rates to site-specific pathways, faster investigation, and demonstrating a broad range of cancer and other serious disease diagnoses through more patient-centred, symptom-driven care.^11^

Managed pathways for those presenting with NSS need to be based on high-quality evaluation, function optimally, and demonstrate sustainability. Making the case for sustainable funding of these pathways can be challenging. Earlier-stage diagnosis may not always be realised, considering that NSS can sometimes be associated with harder-to-diagnose cancers and later-stage disease, and the economic benefit of the pathways has not yet been comprehensively demonstrated. As these pathways embed within the health system, the impact on workload and resourcing must be better understood and planned for.

## Non-specific symptoms: cancer risk

Research investigating NSS has significantly advanced our understanding of both overall and site-specific cancer risk, demonstrating how NSS can enhance risk stratification in primary care. Most research in this space has focused on risk of cancer associated with weight loss, abdominal pain, bloating, and fatigue. Across these symptoms, risk typically increases with age and is generally greater in men than women.

### Unexplained weight loss

The overall cancer risk exceeds 3% in women aged >60 years (4.6%) and in men aged >50 years (4.1%) in patients with unexplained weight loss (UWL). In current or former smokers, risk exceeds 3% in both men and women aged >50 years.^12^ The period of greatest risk of a cancer diagnosis is within the 6 months following the first recorded UWL presentation to primary care.^13^ UWL is most strongly associated with an increased likelihood of pancreatic, cancer of unknown primary, gastro-oesophageal, lymphoma, hepatobiliary, lung, bowel, and renal tract cancer.^13^

In primary care data from the US, the amount of weight lost was more predictive than the amount of time over which it was lost, leading to caution that guidelines should resist giving an arbitrary cut-off for the interval of weight loss and focus on the percentage of weight loss and the patient’s age.^14^ Five per cent of total body weight is regarded as clinically meaningful, warranting further investigation. As weight is infrequently recorded, referral criteria should accept GP concern as patients may present with unquantified UWL expressed as loose fitting clothes or in response to family observation.^13^

### Abdominal pain or bloating

The risk for all cancers does not exceed 3% for either abdominal bloating or pain unless stratified by age.^15^ All cancer risk exceeds 3% in patients aged ≥60 years with either symptom, and increases with age (≥60 years with abdominal pain: men [4.2–8.6%] and women [3.1–5.1%], ≥60 years with bloating: men [3.0–6.7%] and women [3.5–4.6%]). Patients seen in primary care for either symptom had more than double the risk of cancer compared with the general population, with an excess risk observed in all age groups except males aged 30–39 years with bloating.^16^

Site-specific cancer risk does not exceed 1% for either abdominal symptom when considered in isolation but does when considered alongside abnormal blood test results. The most common cancer sites in males experiencing abdominal pain or bloating were colon, prostate, and pancreas, and in females were colon, breast, ovary, and pancreas.^16^ A recent study investigated 1-year cumulative incidence in those with abdominal pain alone for intra-abdominal cancers (oesophago-gastric, colorectal, liver, pancreatic, ovarian, uterine, kidney, and bladder). The risk exceeded 3% in men aged ≥70 years (3.4%), while for women aged ≥70 years it was slightly lower (2.3%). For all age groups, the risk was greatest for colorectal cancer, followed by pancreatic and oesophago-gastric cancers (and ovarian cancer for women).^17^

### Fatigue

The risk of all cancers combined in men aged ≥30 years presenting with fatigue for the first time is reported as 2.6%, and 1.4% for women. Excess cancer risk in those presenting with fatigue, compared with those not presenting with fatigue, is reported as 1.5% in men, and 0.5% for women. This increases with age in women, but was generally smaller than for men. This study found fatigue was more likely to be recorded in coded data by GPs in women, although men with recorded fatigue were more likely to be diagnosed with cancer.^18^

The risk of cancer in men who presented with fatigue exceeded 3% in those aged ≥65 years. The risk of cancer in women was generally lower, exceeding 3% in those aged ≥80 years.^19^ Nearly half (47%) of patients diagnosed with cancer within a year after their initial fatigue record were diagnosed in the first 3 months.^19^

The site-specific risk of cancer does not exceed 3% for men or women with fatigue. However, site-specific cancer risk was higher than expected (compared with general population expected rates) for 13 cancer sites. Leukaemia, pancreatic, and brain cancer were over-represented among patients with fatigue, but, as they are relatively rare cancers, the most common cancers in those with fatigue reflected those in the general population (breast, lung, colorectal, bowel).^19^

Figure 1 outlines risk of all cancers in those with unexplained weight loss, abdominal pain, bloating, and fatigue in combination with other symptoms.^12,15,20^

**Figure 1.**
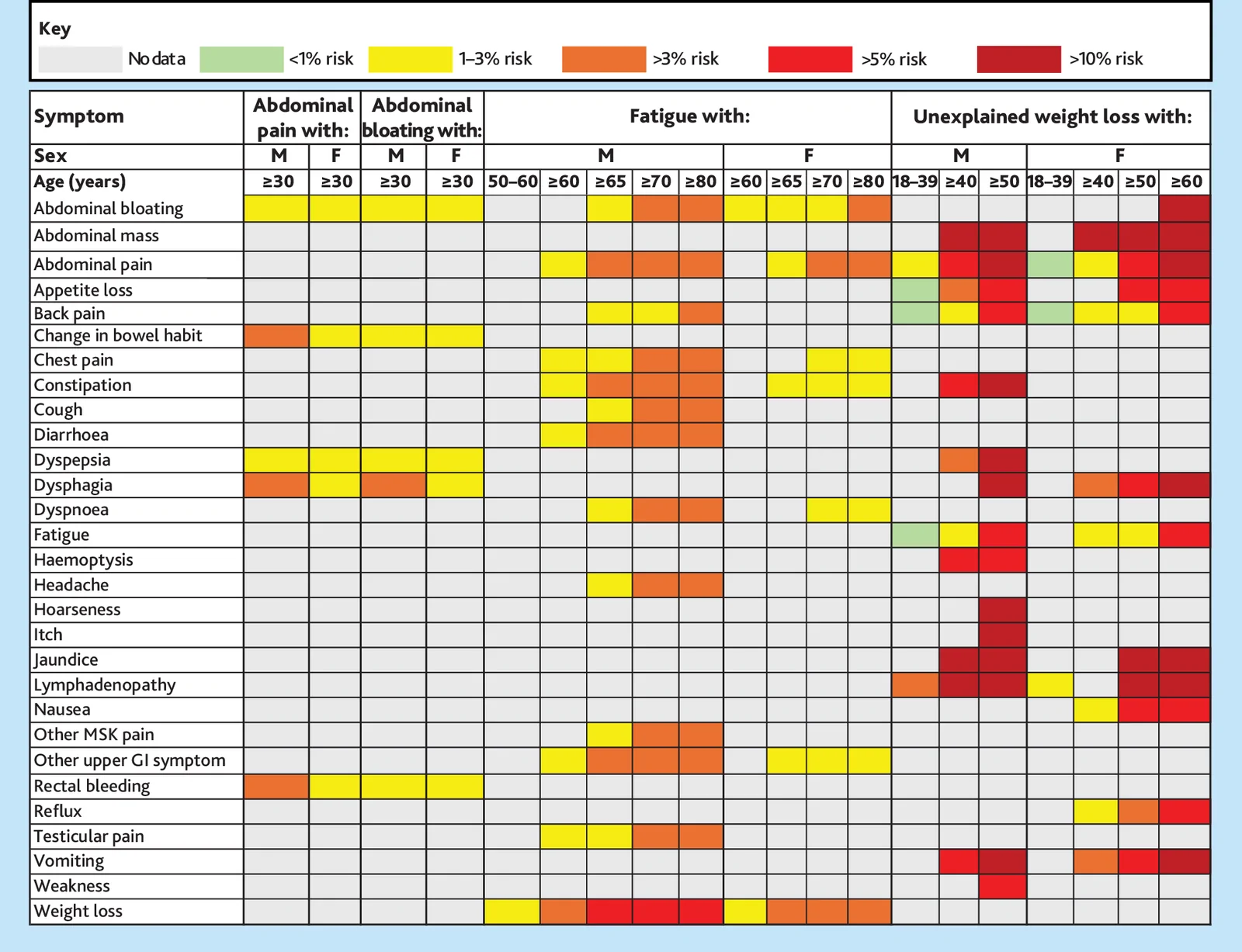
All cancer risk in those presenting with either unexplained weight loss, abdominal symptoms, or fatigue, in combination with other symptoms, by age and sex.^a a^Estimates for weight loss and fatigue combination vary because of studies using varying approaches to estimating population and individual cancer risk over different follow-up periods. Symptom combinations included in the fatigue column are specifically in patients with no red-flag symptoms or anaemia. This may mean the reported risk is lower compared with cancer risk in all patients with fatigue. GI = gastrointestinal. MSK = musculoskeletal. Copyright Cancer Research UK. Used with permission.

When interpreting the findings, it is worth reflecting on some of the common methodological limitations of the included studies.

This research uses electronic health records, so the risk estimates relate to patients with the symptoms who have presented to their GP. Risk estimates among the broader population who experience the same symptom may be lower than the ones reported among presenters. As free text is not available for analysis, estimates are based on coded symptoms only, which could lead to less accurate risk estimates. Additionally, the studies included in Figure 1 have not provided a clear comparison of excess cancer risk, compared with the age–sex specific baseline cancer risk.

## Future directions of NSS management

NSS demonstrate significant all-cancer risk, especially when stratified by age. There is a clear need to provide managed routes to diagnosis for this patient group.

### Optimising current pathways

There are lots of opportunities to improve current practice across the UK that need to be realised, such as optimising NSS pathways and national cancer referral guidelines. Box 1 outlines which NSS individually and in combination may warrant urgent suspected cancer referral and investigation at a risk threshold of 3% that are not currently included in UK national referral guidelines. Modelling of the health system impact of investigating these additional presentations would be beneficial. The risk of over-testing driven by high baseline cancer risk in older patients, and how commonly the symptom presents, has been considered in the recommendations.

**Box 1.** Non-specific symptom referral guideline recommendations

**Weight loss** Weight loss (>5% body weight) in women aged ≥60 years, and men aged ≥50 years. Weight loss, in men in combination with: lymphadenopathy Weight loss in those aged ≥40 years in combination with: vomiting Weight loss, in men aged ≥40 years in combination with: abdominal pain appetite loss constipation dyspepsia Weight loss, in women aged ≥50 years in combination with: abdominal pain appetite loss nausea reflux lymphadenopathy Weight loss, in men aged ≥60 years in combination with: fatigue Weight loss, in women aged ≥70 years in combination with: fatigue **Abdominal pain or bloating** Abdominal pain or bloating in those aged>≥60 years. Abdominal pain, in men aged ≥30 years, in combination with: rectal bleeding change in bowel habit Abdominal pain, in men aged ≥70 years in combination with: fatigue Abdominal pain or bloating, in women aged ≥80 years in combination with: fatigue **Fatigue** Fatigue in men aged ≥70 years Fatigue, in men aged ≥60 years in combination with: weight loss Fatigue, in men aged ≥70 years in combination with: abdominal pain constipation Fatigue, in women aged ≥70 years in combination with: weight loss Fatigue, in women aged ≥80 years in combination with: abdominal pain or bloating

In the future, as pathways evolve and new evidence emerges, modified approaches to existing pathways may be required to better capture the clusters of cancers and other serious diseases diagnosed in patients with NSS. One-stop clinics aim to streamline the diagnostic process, by carrying out a range of tests at the same location. These clinics have been shown to decrease time from referral to testing, with high patient and clinician acceptability.^21^ A pilot project in Australia is ongoing to develop a gastrointestinal symptom guide to support the optimal care pathways, capturing a range of abdominal symptoms and blood test results that overlap with multiple cancer types. These pathways highlight the potential for more innovative, cross-specialty approaches to cancer diagnosis.

## Emerging innovations

New research, tests, tools, and data analytic approaches are shaping the future landscape of managing NSS. To implement these innovations effectively, healthcare systems must be prepared to adopt them. This includes updating digital infrastructure to support more complex analysis of routine data.

The Blood Test Trend for Cancer Detection (BLOTTED) studies are generating insights into whether blood test trends might support earlier cancer detection in patients with NSS.^22^ There are also opportunities to incorporate clinical activity and prescription data alongside investigations, patient presentation, and demographics. The development of novel tests and biomarkers, such as multi-cancer tests based on analysis of circulating tumour DNA, can also complement existing investigations. The SYMPLIFY study^23^ suggests a promising use case for multi-cancer testing could be investigating those presenting with NSS that represent a wide range of cancer types in primary care, to inform decisions to refer, and perhaps save many patients from unnecessary cancer investigation. A large prospective study of multiple multi-cancer tests (ARMADILO) is aiming to accelerate progress by streamlining the evidence generation pipeline for symptomatic use cases.

Figure 2 outlines how innovations could transform NSS pathways in the future.

**Figure 2.**
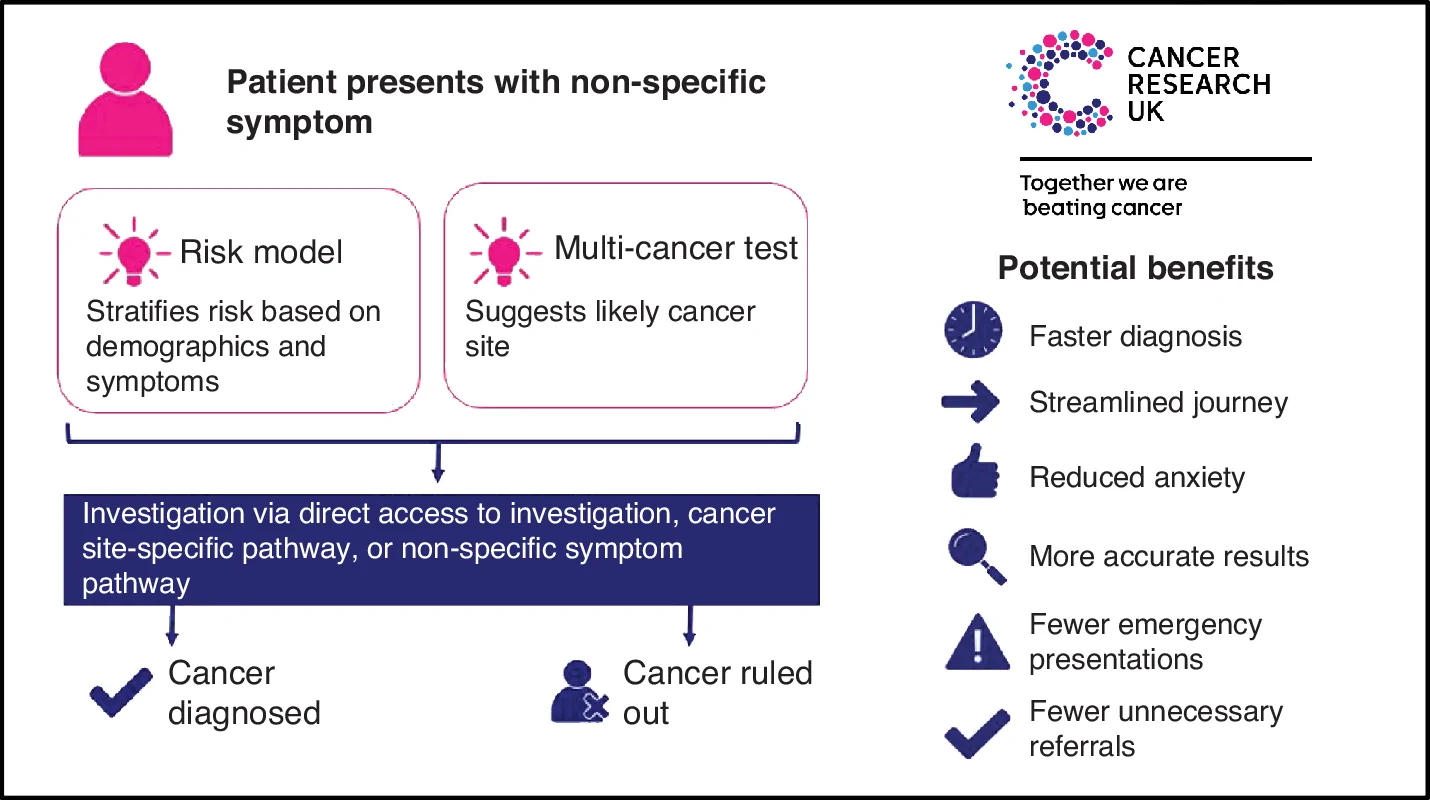
Potential benefits of innovations as part of NSS pathways in the future. NSS = non-specific symptoms. Copyright Cancer Research UK. Used with permission.

To realise these opportunities, further research is needed to improve earlier diagnosis and patient outcomes, including:

- evidence generation to better calculate symptom risk for other NSS, such as appetite loss, nausea, or back or bone pain;
- investigation of the accuracy of existing and novel tests used for patients presenting with NSS in primary care, and the impact on management and referral decisions;
- exploration of what constitutes optimal functioning of NSS pathways to facilitate more timely diagnosis of cancer;
- assessment of the health economics and patient outcomes of the varied delivery models of NSS pathways based on real-world NHS data and long-term follow-up; and
- investigation of the benefits, pitfalls, and implementation challenges of more innovative models, outlining what impact this could have on patients and health systems.

## Conclusion

Significant progress has been made in understanding cancer risk associated with NSS. Research has clarified how factors such as age, sex, and symptom combinations influence risk, emphasising the importance of timely referral even when traditional alarm symptoms are absent. Emerging innovations, including multi-cancer tests and risk stratification modelling, have the potential to transform NSS management, but many of these are not ready for implementation. Adaptations to national referral guidelines and pathways may be a more opportune approach to improve management, leading to timelier and earlier diagnosis of cancer.
